# Terra Firma-Forme Dermatosis Presenting With Unusual Features: A Case Report

**DOI:** 10.7759/cureus.100940

**Published:** 2026-01-06

**Authors:** Meshari A Alalyani, Khuzama M Alaseeri, Shaden O Alqurashi, Khalid Al Hawsawi

**Affiliations:** 1 College of Medicine, King Saud bin Abdulaziz University for Health Sciences, Jeddah, SAU; 2 College of Medicine, Taif University, Taif, SAU; 3 Dermatology, King Abdulaziz Hospital, Makkah, SAU

**Keywords:** asymptomatic verrucous plaque, benign cutaneous disorder, lamellar hyperkeratosis, retention hyperkeratosis, terra firma-forme dermatosis

## Abstract

Terra firma-forme dermatosis (TFFD), also known as retention hyperkeratosis, is a benign cutaneous disorder characterized by dirt-like, brownish patches or plaques that are resistant to conventional hygiene practices, including washing with soap and water. It is due to delayed keratinocyte maturation and retention of melanin and sebum within a compact stratum corneum. Here we report a 25-year-old male who presented with a six-month history of a persistent, asymptomatic, gradually enlarging skin lesion over his chest. Skin examination revealed a large well-demarcated verrucous yellowish plaque on his chest. Clinical differential diagnoses included terra firma, dermatatitis neglecta, confluent and reticulated papillomatosis of Gaugorut and Cartuad (CARP), late onset epidermal nevus, verrucous xanthoma and acanthosis nigricans. A diagnostic wipe test using 70% isopropyl alcohol led to immediate and near-complete resolution of the plaques, supporting the diagnosis of terra firma-forme dermatosis. A punch biopsy was performed prior to doing the diagnostic wipe test with alcohol. It revealed lamellar hyperkeratosis, orthokeratotic, papillomatosis, acanthosis, with no significant dermal inflammation, findings consistent with TFFD.

## Introduction

Terra firma-forme dermatosis (TFFD) is a benign cutaneous disorder first described in 1987, named for its resemblance to “dry land” or “firm earth” [1]. It is also known as retention hyperkeratosis and is most frequently observed on the face, neck, and trunk, particularly in children [2]. Clinically, TFFD presents as asymptomatic, dirt-like, slightly papillomatous, brownish patches or plaques that are resistant to conventional hygiene practices, including washing with soap and water [2,3]. However, these lesions can be easily cleared by rubbing with 70% ethyl or isopropyl alcohol-soaked gauze, which serves as both a diagnostic and therapeutic test [3]. Although the precise etiology remains unclear, it is hypothesized that it results from delayed keratinocyte maturation and retention of melanin and sebum within a compact stratum corneum [3]. While the diagnosis of TFFD is primarily clinical, histopathological examination can rule out other differential diagnosis when diagnosis is in doubt [4]. The limited clinical familiarity with TFFD likely contributes to its significant underdiagnosis [5]. In this report, we describe a distinctive case of TFFD in a 25-year-old male who presented with extensive verrucous plaques over the anterior chest, underscoring the importance of recognizing the broad spectrum of clinical presentations associated with this condition.

## Case presentation

**Figure 1:**
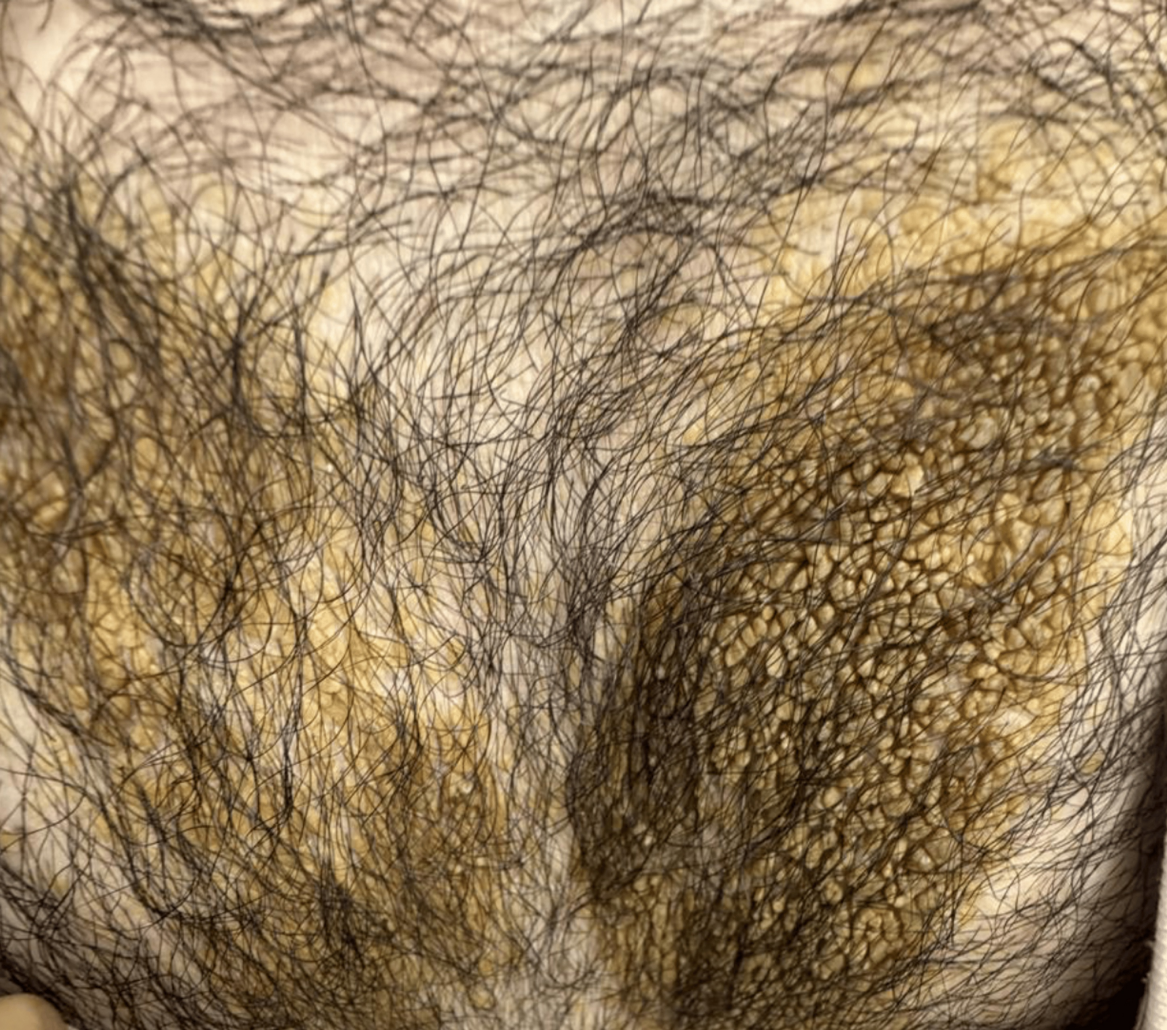
Large well-demarcated verrucous yellowish plaque on the chest wall

A 25-year-old medically free male presented to our outpatient dermatology clinic with a history of asymptomatic, gradually enlarging skin lesion over the chest, persisting for approximately six months. He reported no prior systemic or dermatologic conditions, denied any significant family history, and was otherwise in good general health. Cutaneous examination revealed a large well-demarcated verrucousyellowish plaque on his chest (Figure 1).

**Figure 2:**
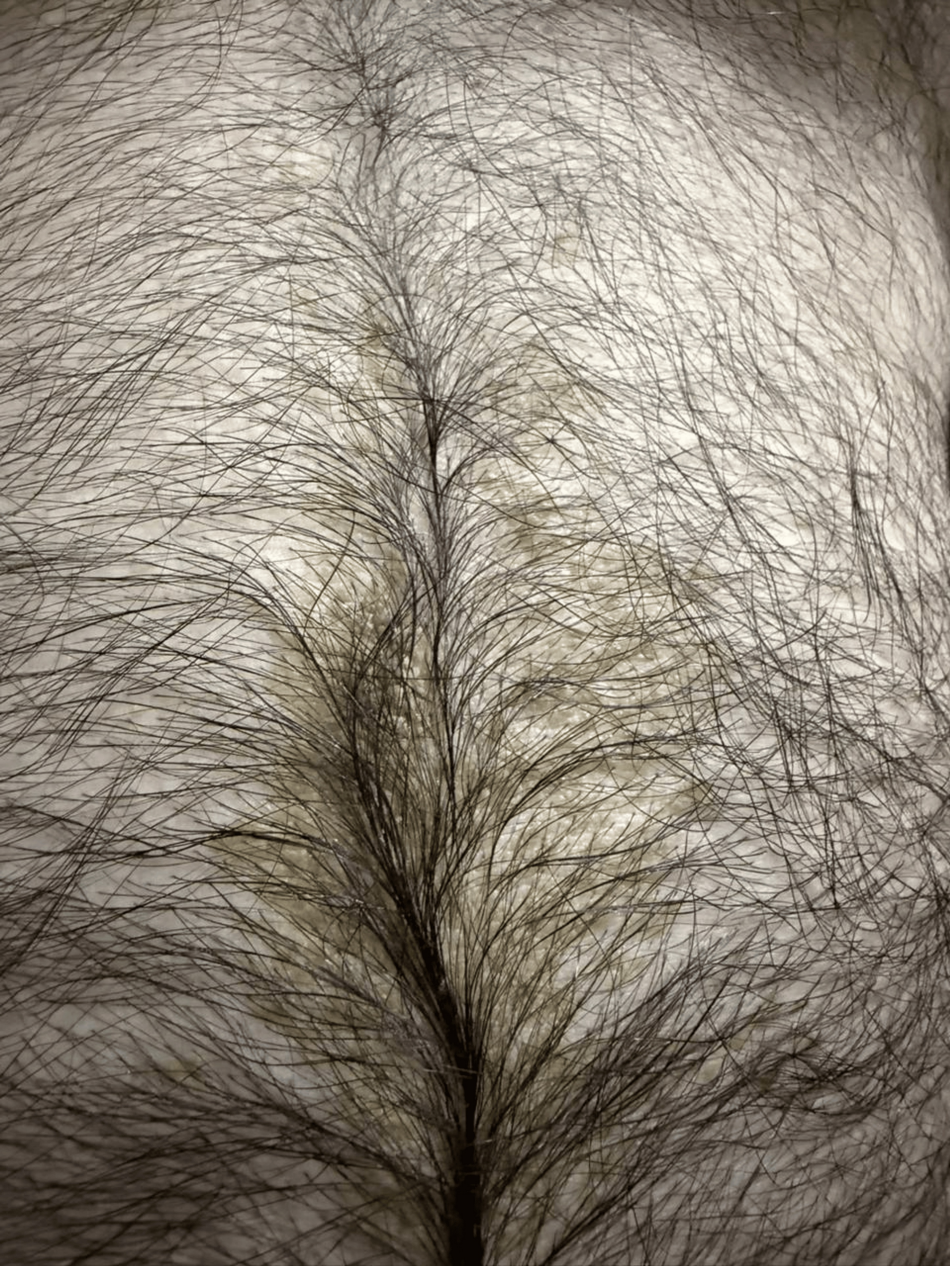
Diagnostic wipe test using 70% isopropyl alcohol led to immediate and near-complete resolution of the plaques

**Figure 3:**
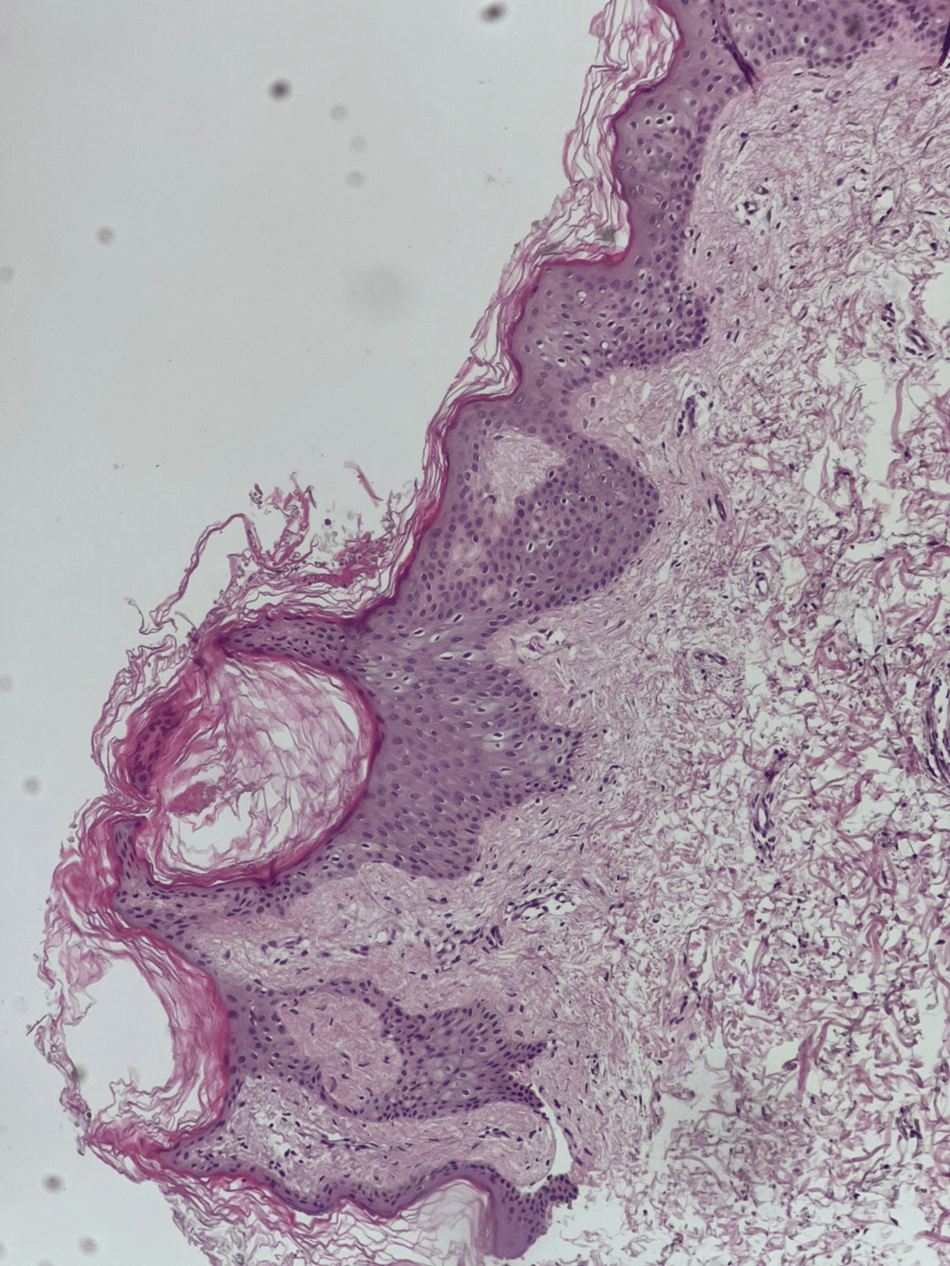
Histopathology of skin lesion showing lamellar hyperkeratosis, orthokeratotic, papillomatosis, acanthosis, with no significant dermal inflammation

No other skin lesions elsewhere on the body. Hair, nail and mucous membrane examination was normal. Clinical differential diagnoses includes terra firma, dermatatitis neglecta, confluent and reticulated papillomatosis of Gaugorut and Cartuad (CARP), late onset epidermal nevus, verrucousxanthoma and acanthosis nigricans. A diagnostic wipe test using 70% isopropyl alcohol led to immediate and near-complete resolution of the plaques (Figure 2), supporting the diagnosis of TFFD. A punch biopsy was performed prior doing the diagnostic wipe test with alcohol. It revealed lamellar hyperkeratosis, orthokeratotic, papillomatosis, acanthosis, with no significant dermal inflammation (Figure 3), finding consistent with TFFD. The patient was reassured about the benign nature of the condition and advised to maintain regular gentle cleansing and exfoliation, with follow-up planned to ensure complete clearance.

## Discussion

The diagnosis of TFFD is often challenging, as it may clinically mimic several dermatoses, including pityriasis versicolor, frictional melanosis, acanthosis nigricans, dermatosis neglecta (DN), confluent and reticulated papillomatosis, and macular amyloidosis [6]. Among these, DN poses the greatest diagnostic dilemma due to its overlapping clinical features. However, a key distinguishing factor lies in the patient’s hygiene history and lesion response to cleansing. DN is typically associated with inadequate hygiene and resolves with soap and water, whereas TFFD occurs in individuals with otherwise normal hygiene and resolves exclusively with vigorous swabbing using isopropyl alcohol [7,8]. Thus, the alcohol wipe test serves as a rapid, cost-effective, and minimally invasive diagnostic and therapeutic tool [7,9]. Adjunctive diagnostic tools may offer additional clarity. Dermoscopy of TFFD characteristically reveals a “mosaic” pattern composed of polygonal brown clods [10]. Wood’s lamp examination may demonstrate chalk-white fluorescence in certain cases, though this finding lacks consistency and is not diagnostic. Importantly, due to the lesion’s resemblance to various dermatoses, patients are often subjected to unnecessary investigations, as was the case in our patient. This diagnostic challenge may be further amplified in patients with skin of color, where increased baseline pigmentation can make the lesions appear more pronounced and raise concern for melanocytic or other pigmented disorders, potentially leading to misdiagnosis and unnecessary invasive investigations. The biopsy was carried out in our patient because we did not consider TFFD in our differential diagnosis because we did not expect to see TFFD with such severe presentation. However, one of the residents attempted the alcohol wipe test with 70% alcohol, which fortunately led to immediate and near-complete resolution of the wiped area. Although the literature remains limited, increasing reports of adult-onset TFFD suggest that the condition may be underrecognized in older populations. Moreover, extensive thoracic involvement, as demonstrated in this case, is particularly rare [11]. Clinician awareness is essential to prevent misdiagnosis and avoid unwarranted interventions such as biopsies, fungal cultures, or systemic therapies. The recurrence of TFFD lesions, when it occurs, is typically responsive to repeat alcohol swabbing, supporting the benign and self-limited nature of this condition. Although TFFD is predominantly reported in children and adolescents, particularly in intertriginous regions such as the neck and axillae, the current case illustrates an atypical presentation in a 25-year-old adult with severe TFFD. This unusual severe TFFD underscores the need for heightened clinical awareness, as TFFD is not confined to pediatric populations nor to have a mild presentation. Recognizing such variability in age of onset and severity of presentation of TFFD is essential to prevent misdiagnosis and avoid unnecessary investigations and treatment.

## Conclusions

This case highlights that TFFD can present in adults with extensive and severe lesions, mimicking other dermatoses such as dermatosis neglecta. Recognition of TFFD and use of the simple alcohol wipe test can prevent unnecessary investigations and interventions. Clinicians should be aware of its variable presentation across age groups and severity to ensure timely and appropriate management.
